# Human Leukocyte Antigen-Allelic Variations May Influence the Age at Cancer Diagnosis in Lynch Syndrome

**DOI:** 10.3390/jpm14060575

**Published:** 2024-05-27

**Authors:** Lutricia Ndou, Ramadhani Chambuso, Ziyaad Valley-Omar, George Rebello, Ursula Algar, Paul Goldberg, Adam Boutall, Raj Ramesar

**Affiliations:** 1 UCT/MRC Genomic and Precision Medicine Research Unit, Division of Human Genetics, Department of Pathology, Institute of Infectious Disease and Molecular Medicine, The University of Cape Town, Affiliated Hospitals, Cape Town 7704, South Africa; 2 Medical Virology, National Health Laboratory Service, Groote Schuur Hospital, The University of Cape Town, Cape Town 7925, South Africa; 3 The Colorectal Unit of the Department of Surgery, Groote Schuur Hospital, The University of Cape Town, Cape Town 7925, South Africa

**Keywords:** Lynch syndrome, cancer risk, age at cancer diagnosis, germline pathogenic variant *MLH1*:c.1528C > T, human leukocyte antigen (HLA), genetic risk modifiers, personalized cancer screening

## Abstract

Lynch syndrome (LS) is an inherited cancer predisposition disorder associated with an elevated risk of developing various solid cancers, but mostly colorectal cancer (CRC). Despite having the same germline pathogenic variant (PV) in one of the mis-match repair genes or the *EPCAM* gene, Lynch syndrome variant heterozygotes (LSVH) exhibit a remarkable phenotypic variability in the risk of developing cancer. The role of human leukocyte antigen (HLA) in modifying cancer development risk prompted our hypothesis into whether HLA variations act as potential genetic modifiers influencing the age at cancer diagnosis in LSVH. To investigate this, we studied a unique cohort of 426 LSVH carrying the same germline PV in the *hMLH1* gene (*MLH1*:c.1528C > T) in South Africa. We intuitively selected 100 LSVH with the greatest diversity in age at cancer diagnosis (N = 80) and the oldest cancer unaffected LSVH (N = 20) for a high-throughput HLA genotyping of 11 HLA class I and class II loci using the shotgun next-generation sequencing (NGS) technique on the Illumina MiSeq platform. Statistical analyses employed Kaplan–Meier survival analyses with log-rank tests, and Cox proportional hazards using binned HLA data to minimize type I error. Significant associations were observed between young age at cancer diagnosis and HLA-DPB1*04:02 (mean age: 37 y (25–50); hazard ratio (HR) = 3.37; corrected *p*-value (q) = 0.043) as well as HLA-DPB1 binned alleles (including HLA-DPB1*09:01, HLA-DPB1*10:01, HLA-DPB1*106:01, HLA-DPB1*18:01, HLA-DPB1*20:01, HLA-DPB1*26:01, HLA-DPB1*28:01, HLA-DPB1*296:01, and HLA-DPB1*55:01) (mean age: 37 y (17–63); HR = 2.30, q = 0.045). The involvement of HLA-DPB1 alleles in the age at cancer diagnosis may highlight the potential role of HLA class II in the immune response against cancer development in LSVH. When validated in a larger cohort, these high-risk HLA-DPB1 alleles could be factored into cancer risk prediction models for personalized cancer screening in LSVH.

## 1. Introduction

Lynch syndrome (LS) is an autosomal-dominantly inherited cancer-predisposing disorder caused by a germline pathogenic variant (PV) in one of the mismatch repair (MMR) genes or deletions in the 3′ region of the *EPCAM* gene [1]. LS is associated with a very high lifetime risk of developing primarily colorectal cancer (CRC) and extracolonic cancers at a younger age, compared to the general population [1,2,3]. The lifetime risk of developing cancer in LS variant heterozygotes (LSVH) ranges from 30% to 80% depending on the mutated gene, cancer type, and other factors such as lifestyle, environmental exposure, epigenetic changes, and genetic risk modifiers [4,5,6]. This lifetime risk of developing cancer in LSVH differs in terms of age of cancer diagnosis (often used as a proxy for age of onset) and tumor site, even among individuals carrying the same PV [7,8,9,10]. Thus, the identification of additional specific genetic risk modifiers contributing to phenotype variations in cancer risk and age at cancer diagnosis in LSVH may assist in the implementation of highly personalized surveillance and screening interventions to reduce morbidity and mortality related to cancer in this at-risk population.

In LS cancer microenvironments, carcinogenesis is driven by immunoediting through the counter-selection of cell clones presenting frameshift neoantigens (produced as a consequence of MMR deficiency), which depends mainly on the human leukocyte antigen (HLA) alleles [9,11]. HLA alleles are responsible for presenting cellular antigens and eliciting antigen-specific immune responses [12]. While exogenous antigens are presented by HLA class II molecules, endogenous antigens are presented by HLA class I molecules, which interact with CD4-positive and CD8-positive T cells, respectively, which are among the most powerful mediators of anti-tumor immune responses [9,13,14]. HLA alleles are highly diverse among different individuals and populations. Each HLA allele has a unique shape and chemical properties in its antigen-binding groove, which enables it to fit with a specific antigen to a varying degree [9,15,16,17]. Thus, an individual’s HLA typing is crucial in determining the binding of the antigens for presentation on the surface of the cell and eliciting antigen-specific immune response. However, it is not clear whether HLA allele variations may protect or influence cancer initiation in LSVH, hence the variability in cancer risk and age of cancer diagnosis/onset in these individuals [9]. This is because the crucial presentation of cancer antigens to the immune system by the HLA alleles may have a positive or negative impact on tumor initiation and progression, which can modify an individual’s cancer onset risk [9,11,13,18,19]. For example, effective presentation of cancer antigens by HLA to the immune system has been found to play a significant role in the effectiveness of cancer immunotherapy, which aims to reactivate the impaired HLA-mediated anti-tumor immune response, such as immune checkpoint blockade [20,21].

The unique characteristics of cancer pathogenesis in LSVH present an opportunity to study the possible influence of HLA variations on cancer risk and the age of cancer onset. By investigating the impact of HLA allele variations on cancer incidence, cancer onset, and mutation profile in LSVH, we can gain new insights into the role of HLA alleles as modifiers of cancer risk. Several studies have investigated the influence of HLA allele variations on cancer susceptibility [22,23,24,25,26]. However, none of these studies investigated the influence of HLA allele variations on cancer risk or the age at cancer diagnosis in LSVH. We hypothesize that HLA allele variations may influence an individual’s age at cancer diagnosis in LSVH.

In this exploratory study, we investigated our hypothesis using a unique cohort of LSVH carrying the same germline PV in the *hMLH1* gene (NM_000249.4(*MLH1*):c.1528C > T (p.Gln510Ter)) in South Africa.

## 2. Materials and Methods

### 2.1. Patients

**Figure 1:**
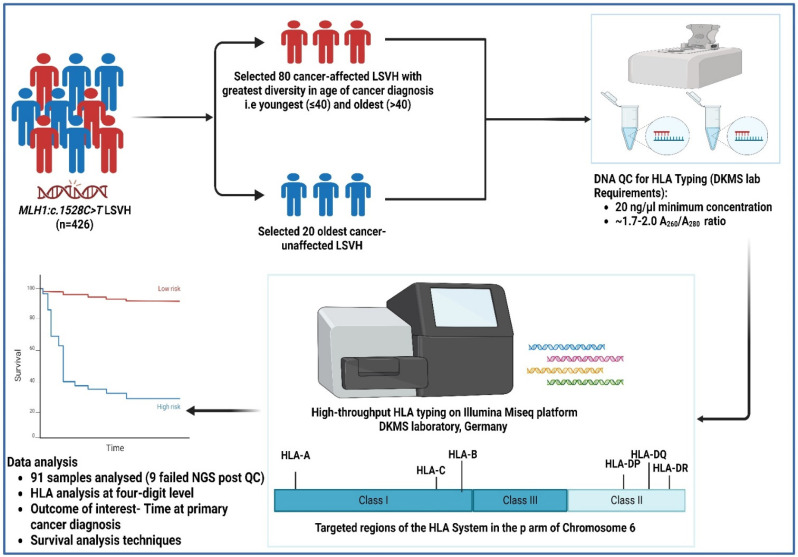
A schematic illustration of the study methodology.

In our large homogenous cohort of 426 genetically confirmed LSVH carrying the same *MLH1*:c.1528C > T South African founder PV, we selected 100 subjects from 40 independent families for this study based on the following inclusion criteria: (i) patients exhibiting the greatest diversity in age at cancer diagnosis, i.e., patients who had 3SD below (youngest) and above (oldest) the mean age at cancer diagnosis from both extremities (N = 80; mean age 42.9; SD ± 11.1 years); (ii) the oldest individuals who were not yet affected with cancer (N = 20) (in order to gauge whether there may be a protective effect across the HLA system); and (iii) the availability of blood genomic DNA sample with a minimum concentration of 20 ng/µL in our designated biorepository. All cancer patients were confirmed through pathology reports indicating the gender, age at the time of first diagnosis, presence or absence of malignancy, and the tumor site. Other demographics, such as ethnicity, were retrieved from our in-house LS electronic database (Figure 1).

### 2.2. DNA Samples

DNA samples of subjects meeting our inclusion criteria were retrieved from the −80 °C biorepository at the Division of Human Genetics, University of Cape Town, South Africa. Genomic DNA was extracted from white blood cells (buffy coats). The DNA was quantified using a NanoDrop spectrophotometer and viewed using version 3.8.1 of the Nanodrop 1000 operating software program (Thermo Fisher Scientific™, Johannesburg, South Africa). The integrity of the DNA samples was checked using 2.0% (*w*/*v*) gel electrophoresis, and the gel was visualized using the UVIpro Gold Transilluminator and through UVPro version 12.3 software (UVItec, Cambridge, UK). DNA samples with the A_260_/A_280_ ratio of 1.7–2.0 and a minimum of 20 ng/ul concentration were selected for the downstream HLA high-throughput typing as per requirement by the Deutsche Knochenmarkspenderdatei (DKMS) laboratory, Germany (Figure 1).

### 2.3. HLA Typing

High-throughput HLA genotyping targeting a total of eleven (11) HLA class I (A, B, and C) and class II (DRB1, DRB3, DRB4, DRB5, DQA1, DQB1, DPA1, and DPB1) loci was performed at the DKMS Life Science Lab, Germany. The shotgun next-generation sequencing (NGS) technique was used on the Illumina MiSeq platform (Illumina, San Diego, CA, USA). All laboratory procedures and the sequencing strategy were performed similarly to a previous publication [27]. Nine (9) out of hundred (100) samples failed the quality control (QC) measure for NGS and were excluded from the downstream analysis. Due to a small sample size, we limited our downstream analysis to a four-digit level to investigate the influence of HLA alleles on the age at cancer diagnosis, as the amino acid sequence of the HLA protein distinguishes the biological effects of different HLA alleles [28] (Figure 1). The potential novel allele in the *DRB3* locus was characterized using the NGS-engine NGS-HLA typing software package (Version 2.15, GenDX) and the IPD-IMGT/HLA database (Version 3.54) [29].

### 2.4. Statistical Analysis

Statistical analysis was performed using R statistical software (R Core Team, version 4.3.3). The outcome of interest was time at first cancer diagnosis (i.e., either CRC or extracolonic cancer). The risk of cancer diagnosis in one group relative to a reference group, at any age, was calculated using survival analysis techniques (Kaplan–Meier product limit method, with log-rank tests and Cox proportional hazards with 95% confidence intervals (CIs)), taking into account the fact that our research cohort included subjects who were cancer-unaffected. Continuous data were presented as mean and standard deviation (SD) or median and interquartile range (IQR), whereas categorical data were presented as numbers (percentage). To reduce the risk of type I errors in our statistical analysis caused by the high variability of alleles in the HLA region, we grouped rare alleles (those with counts < 5) together (binning) at each locus. After this process, we had a total of 20 HLA-A, 14 HLA-B, 10 HLA-C, 11 HLA-DRB1, 5 HLA-DRB3, 3 HLA-DRB4, 3 HLA-DRB5, 9 HLA-DQA1, 10 HLA-DQB1, 8 HLA-DPA1, and 12 HLA-DPB1 alleles. The immunotation R package (Version 1.10.0) was used to call the frequencies of HLA alleles from the IPD-IMGT/HLA Database (Version 3.54) [29,30]. All tests were two-tailed, and *p* values were corrected for multiple comparisons according to the Benjamini–Hochberg method. Associations were considered significant if both the *p*- and q-values (adjusted *p*-value) were <0.05 (Figure 1).

## 3. Results

### 3.1. Overall Demographic and Clinical Characteristics of the Patients

Demographics and clinical characteristics of two groups (cancer-affected and cancer-unaffected) of LSVH carrying the same PV (*MLH1*:c.1528C > T) in the *hMLH1* gene are summarized in Table 1. There were 78 cancer-affected and 13 cancer-unaffected LSVH. Of the 78 cancer-affected patients, 60 (78%) were diagnosed with CRC. As expected in LS, proximal colon tumors were the most common (58%) in this cohort (Table 1).

### 3.2. Effects of Gender and Cancer Type on Age at Cancer Diagnosis and Incidence Rates in LSVH

**Figure 2:**
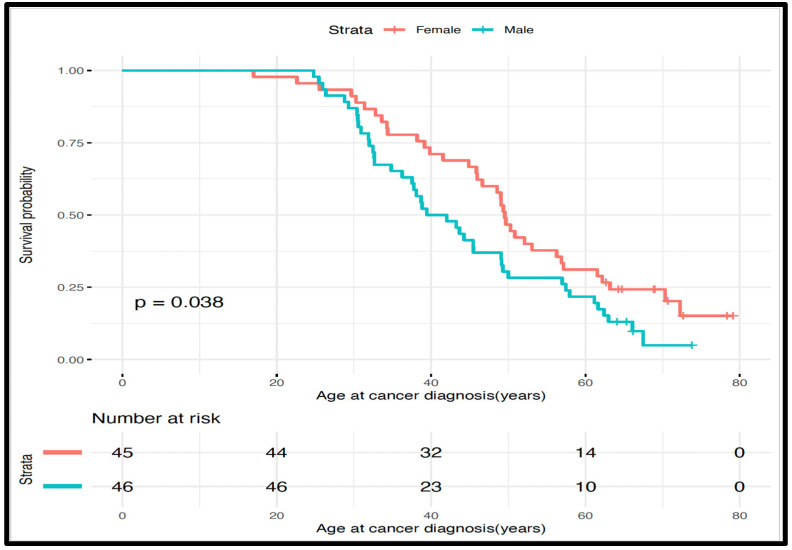
Kaplan–Meier plots illustrating a significant difference in trends of age at cancer diagnosis in LSVH stratified by gender showing that male patients developed cancer significantly earlier than female patients.

To find out whether the trends of age at cancer diagnosis overtime were influenced by the gender of an individual in this cohort, we used Kaplan–Meier survival analysis taking into account that there were individuals who were not yet affected. We used age at cancer diagnosis as our outcome of interest to determine whether there is a difference in trends of age at cancer diagnosis overtime between male and female patients. We found that male LSVH had significant trends of younger age at cancer diagnosis compared to female LSVH (mean age: 40.7 y (95% CI: 37.5–49.1) and 49.6 y (95% CI: 46.0–57.1), respectively, *p* = 0.038), while female patients exhibited a consistently higher survival probability (Figure 2). Our observations are consistent with previous findings on the effect of gender on cancer risk/incidence in LSVH [31,32,33]. Therefore, gender was considered a confounding factor in this cohort.

**Figure 3:**
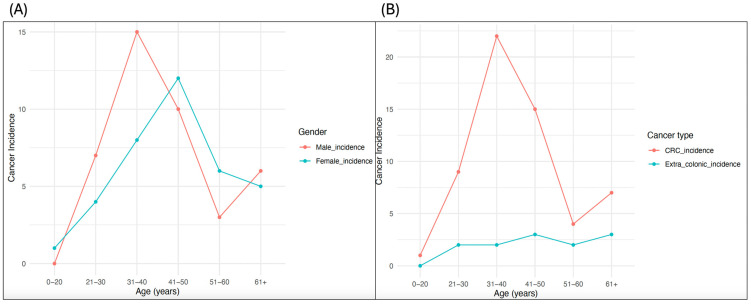
Cancer incidence plots showing comparisons across different age groups in LSVH stratified by (**A**) gender and (**B**) cancer type.

Also, the incidence of cancer was higher in male patients than in female patients (Figure 3A), while CRC incidence was the highest across all age groups compared to extracolonic cancers in this cohort (Figure 3B). These findings were also similar to other cohorts of LSVH carrying different PVs in that the risk of developing CRC was typically higher and at a younger age compared to most extracolonic cancers [34,35].

### 3.3. Effects of HLA Alleles on the Age at Cancer Diagnosis in LSVH

In order to study the effects of HLA allele variations on the age at cancer diagnosis in this cohort, we performed a Cox regression analysis to investigate whether HLA allele variations can influence age at cancer diagnosis in LSVH. We further adjusted our analysis by gender to avoid the potential confounding effects of gender bias. Cumulatively, we identified 31 alleles in HLA-A, 38 in HLA-B, 21 in HLA-C, 28 in HLA-DRB1, 6 in HLA-DRB3, 3 in HLA-DRB4, 3 in HLA-DRB5, 11 in HLA-DQA1, 14 in HLA-DQB1, 9 in HLA-DPA1, and 20 in HLA-DPB1 (Supplementary Table S1). Due to low counts of certain alleles (highlighted in blue in Supplementary Table S1), we grouped 12, 25, 12, 18, 2, 3, 5, 2, and 9 alleles from HLA-A, HLA-B, HLA-C, HLA-DRB1, HLA-DRB3, HLA-DQA1, HLA-DQB1, HLA-DPA1, and HLA-DPB1, respectively, into specific categories (HLA-A binned, HLA-B binned, HLA-C binned, HLA-DRB1 binned, HLA-DRB3 binned, HLA-DQA1 binned, HLA-DQB1 binned, HLA-DPA1 binned, and HLA-DPB1 binned) before conducting the Cox regression analysis. This adjustment resulted in 20 alleles for HLA-A, 14 for HLA-B, 10 for HLA-C, 11 for HLA-DRB1, 5 for HLA-DRB3, 3 for HLA-DRB4, 3 for HLA-DRB5, 9 for HLA-DQA1, 10 for HLA-DQB1, 8 for HLA-DPA1, and 12 for HLA-DPB1 alleles.

Table 2 presents the Cox regression findings of HLA class I (HLA-A, B, and C) and HLA class II (DRB1, DRB3, DRB4, DRB5, DQA1, DQB1, DPA1, and DPB1) in 78 LSVH with cancer and 13 LSVH without cancer. The statistical analysis, including the binned groups, resulted in some significant HLA class II allele associations. Specifically, HLA- DPB1*04:02 (HR = 3.37, *p* = 0.01, q = 0.043) and HLA-DPB1 binned alleles (including HLA-DPB1*09:01, HLA-DPB1*10:01, HLA-DPB1*106:01, HLA-DPB1*18:01, HLA-DPB1*20:01, HLA-DPB1*26:01, HLA-DPB1*28:01, HLA-DPB1*296:01, and HLA-DPB1*55:01) (HR = 2.30; *p* = 0.01; q = 0.045) were significantly associated with a younger age at cancer diagnosis (Table 2). There was no statistically significant association between all other HLA alleles observed and the age at cancer diagnosis in this cohort, as shown in Table 2. Interestingly, a potential novel allele in the DRB3 locus was detected in our youngest cancer patient diagnosed at the age of 17 years. This potential novel allele bearing the HLA-DRB3:g.7953C > T variant is most similar to the HLA-DRB3*03:01 allele (Supplementary Figure S1).

### 3.4. Different HLA Allele Frequencies between LSVH and the Previously Studied South African General Populations

South Africa has a diverse population with a unique genetic background, (ranging from indigenous African subpopulations, and immigrant European and Asian populations) and varying disease prevalence. This diversity could present unique HLA allele variations that are not found in other populations within the country (and internationally) [36,37,38]. Comparing HLA allele frequency (AF) between LSVH and different populations in South Africa allows for us to account for these population-specific factors. This information can aid in tailoring cancer screening and management approaches that are more effective and relevant to the South African context. Also, it can identify specific novel HLA alleles that may be associated with LS susceptibility or be likely protective, and unique amongst this cohort, compared to the general population. This information may contribute to early cancer risk assessments and highly personalized prevention strategies for LSVH in South Africa [39].

**Figure 4:**
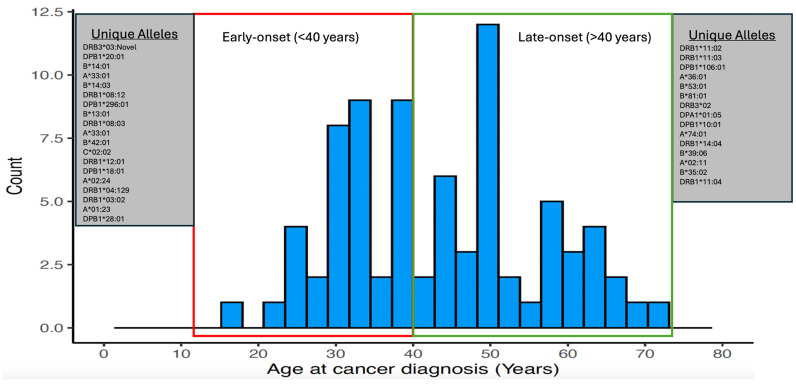
A histogram showing the distribution of age at cancer diagnosis and unique HLA alleles observed in both extremes.

**Figure 5:**
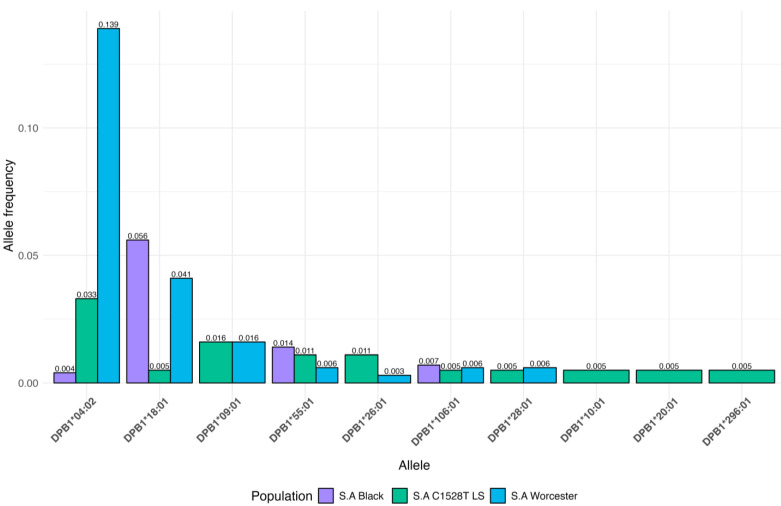
Comparison of HLA allele frequencies of the HLA-DPB1*04:02 allele and *HLA-DPB1* rare alleles in the binned category associated with young age at cancer diagnosis in our LSVH cohort (sea green) and the previously studied South African general population (purple and blue).

Figure 4 shows unique alleles (i.e., alleles that were only observed once in the LSVH cohort) in both extremes of age at cancer diagnosis, as they might provide some explanation for the diverse phenotype observed in these LSVH. We also compared the AF of the HLA alleles that were significantly associated with age at cancer diagnosis and those that were unique in both extremes of age at cancer diagnosis in our LSVH cohort with the AF observed from other non-LS South African cohorts reported in the IPD-IMGT/HLA Database, Figure 5, and Supplementary Table S2, respectively [29]. The HLA-DPB1*04:02 allele was observed with a frequency of 3.3% and 7.5% in the LSVH cohort and the South African general population, respectively **(Figure 5**). Interestingly, the following alleles were observed for the first time in the South African study cohort: HLA-A*02:24, HLA-A*74:01, HLA-B*14:03, HLA-DPA1*01:05, HLA-DPB1*10:01, HLA-DPB1*20:01, HLA-DPB1*296:01, HLA-DRB1*04:129, HLA-DRB1*08:12, and HLA-DRB1*11:03 (Supplementary Table S2).

## 4. Discussion

This is the first study aimed at identifying HLA class I and II alleles that may influence the age at cancer diagnosis in LSVH carrying the same PV in the *hMLH1* gene (*MLH1*:c.1528C > T). Our findings suggest that, once validated in a large cohort, the identification of high-risk HLA alleles could be factored into the risk prediction model calculations for offering tailored personalized cancer screening and surveillance strategies for LSVH.

Our study is part of an ongoing program promoting the utility of personalized early cancer prevention in LSVH. In this instance, the strategy is to consider the effects of HLA allele variations as one of the potential genetic modifiers for cancer onset risk in a well-defined LSVH cohort. Importantly, personalized screening strategies will potentially reduce the overuse of invasive colonoscopies for CRC screening and (premature) cancer-preventive surgeries in LSVH [40,41].

The strongest association with a young age at cancer diagnosis in LSVH was conferred by the presence of the HLA-DPB1*04:02 class II allele (Table 2). The HLA-DPB1*04:02 allele was common in both LSVH cohort and non-LS South African general populations combined, with the reported allele frequencies of 0.033 (3.3%) and 0.075 (7.5%), respectively (Figure 5). Therefore, once validated in a longitudinal study with a large cohort of LSVH, this potentially high-risk HLA allele may be considered as part of cancer risk assessment in LSVH, potentially promoting more genetically informed predictive testing and much more precisely targeted surveillance for cancer prevention strategies in South Africa.

Worldwide in non-LS populations, different HLA alleles have been reported to be associated with various cancers, such as cervical [26,42,43], leukemia [44], hepatocellular [45], lung squamous cell [46], cutaneous T cell lymphoma [47], and gastric carcinomas [48,49]. For instance, the HLA-DP gene polymorphisms (HLA-DPB1*03:01 and HLA-DPB1*13:01) have been significantly associated with an increased risk of cervical cancer in Chinese populations [50,51,52,53]. Furthermore, HLA-DPB1*04:02:01:21 has been recently reported as a novel HLA allele in a patient with acute leukemia in the UK [44]. In our LSVH cohort, the associations between HLA-DP alleles and cancer risk are consistent with previous associations in non-LS populations across various cancers worldwide [44,50,54]. The HLA-DP locus forms part of the highly polymorphic HLA class II molecules, and genetic variations in HLA alleles may lead to variations in the antigen presentation in the specialized antigen-presenting cells, such as dendritic cells, thus potentially influencing or likely protecting against the development of cancer in LSVH [55,56].

Although we found a statistically significant association between rare binned HLA-DPB1 alleles (HLA-DPB1*09:01, HLA-DPB1*10:01, HLA-DPB1*106:01, HLA-DPB1*18:01, HLA-DPB1*20:01, HLA-DPB1*26:01, HLA-DPB1*28:01, HLA-DPB1*296:01, and HLA-DPB1*55:01) and young age at cancer diagnosis, additional investigation for these HLA alleles in a large cohort of LSVH is required to further complement and validate our findings. While HLA class II is traditionally associated with presenting antigens from extracellular pathogens, it also plays a role in presenting tumor antigens [56,57]. It has been reported that HLA class II antigen expression by tumor cells can influence the tumor antigen (TA)-specific immune responses [56]. Furthermore, another potential explanation for the involvement of HLA class II alleles in cancer risk in LS could be related to the presentation of antigens derived from extracellular sources, such as tumor-derived exosomes or antigens released by dying tumor cells. This can trigger immune responses mediated by CD4+ T cells, including activation of cytotoxic T cells and B cells. Additionally, variations in HLA class II alleles may influence the efficiency of antigen presentation and subsequent activation of the immune response against cancer cells. In LSVH, mutations in mismatch repair genes lead to the accumulation of DNA replication errors and the generation of neoantigens, which can activate the immune responses [58]. Further investigation into the specific mechanisms by which HLA class II alleles influence the immune response in LS could provide valuable insights into the interplay between the immune system and cancer development in LS.

Our findings suggest that HLA-allelic (amongst other genomic) variations could be potential factors influencing the age at cancer diagnosis in LSVH individuals [7,59,60,61,62,63,64,65,66]. In this regard, it is worth considering that these findings, once confirmed in a large cohort of LSVH, could be used to implement more precise pre-symptomatic cancer surveillance programs as follows: (i) integrating HLA allele testing into routine cancer screening for LSVH, as those with certain low or high risk HLA alleles may require decreased or increased highly specialized screening and surveillance, respectively; (ii) use of individual HLA allele information to stratify LSVH with the same PV into different risk groups for age at cancer onset (early or late), which could inform the frequency and intensity of their colonoscopic surveillance; (iii) consider HLA-allelic information when deciding which cancer prevention strategy to recommend to LSVH individuals with known PVs, as those with certain high risk HLA alleles may benefit more from specific lifestyle changes or prophylactic treatments such as hysterectomy for endometrial cancer prevention or the use of aspirin and resistant starch for CRC prevention [41,67]; (iv) develop predictive mathematical models that could take into account of both PVs and different HLA alleles to estimate an individual’s lifetime risk of developing cancer and the likely estimated age at cancer diagnosis in LSVH. However, HLA-allelic variations could be just one of many genetic modifiers that can influence a person’s risk of developing cancer in LSVH. Other already known cancer risk modifiers can affect the overall cancer risk in LSVH. These risk modifiers include polymorphisms in xenobiotic metabolism genes, epigenetic changes, lifestyle factors, and strong family history of cancer [7,59,60,61,62,63,64,65,66].

The strengths of our study include the following: (a) it is the first research study to investigate the associations between HLA class I and II alleles and the age at cancer diagnosis using a genetically confirmed LS cohort; (b) the utilization of high-throughput HLA genotyping using NGS in this regard; (c) the homogenous nature of the study cohort as patients harbor the same LS-PV in the *hMLH1* gene and are originating from a population of a common ethnicity; and (d) new evidence suggesting that HLA allele variations may influence the age at cancer diagnosis in LSVH. The main limitations of our study are (i) the relatively small sample size, mainly because this was an exploratory study and financial constraints to perform high-throughput NGS-based HLA typing in the whole cohort of LSVH; and (ii) we only performed a cross-sectional association study in LSVH without taking into account other possible cancer risk genetic and epigenetic modifiers, cofounders, and the proved causality (causal effect relationship). However, our results suggest a direction for additional investigation and hypothesis generation, which is a critical role for studies in emerging or niche fields. We are making efforts to perform HLA typing in a large longitudinal cohort of LSVH to overcome these limitations in our next study.

## 5. Conclusions

This preliminary study provides valuable insights into the potential role of HLA allele variations and the age at cancer diagnosis in LSVH. HLA-allelic variations may influence the age at cancer diagnosis in LSVH carrying the same germline PV in the *hMLH1* gene (*MLH1*:c.1528C > T). When confirmed in a large longitudinal cohort, it could be worth considering HLA allele information when recommending personalized cancer prevention strategies for LSVH. Targeted HLA typing for high-risk alleles can be included in LSVH routine cancer screening programs and prediction models for personalized cancer screening. This can be achieved by stratifying these individuals into different risk groups based on their at-risk HLA alleles.

## Figures and Tables

**Table 1:** Demographic and clinical characteristics of LS carriers investigated in this study.

Variable	Cancer-Unaffected Controls (LS Carriers) (N = 13)	Cancer-Affected Cases (N = 78)		Total Subjects (N = 91)	* *p*-Value
		Early Diagnosis (≤40) (N = 35)	Late Diagnosis (>40) (N = 43)		
**Gender**					
Male	4 (30.8%)	23 (65.7%)	19 (44.2%)	46	0.057
Female	9 (69.2%)	12 (34.3%)	24 (55.8%)	45	
* Age at diagnosis (years)					
Mean (SD)	69.2 (5.44)	31.9 (5.24)	53.1 (8.31)		
Median (min, max)	68.8 (62.7, 79.1)	32.0 (17.0–39.4)	50.0 (39.8–72.3)		
**Tumor site**					
Proximal colon	NA	24	21	45	0.080
Distal colon	NA	5	5	10	0.726
Rectum	NA	2	1	3	0.441
Endometrium	NA	0	8	8	N/A
Breast	NA	0	3	3	N/A
Ovary	NA	1	0	1	N/A
Small intestine	NA	2	3	5	0.818
Bladder	NA	1	0	1	N/A
Kidney	NA	0	1	1	N/A
Skin	NA	0	1	1	N/A

Notes: * *p*-value for the difference between cancer cases with early diagnosis (≤40) and late diagnosis (>40) calculated using a Z-score test for two proportions with a two-tailed significance level of 0.05. * Age at cancer diagnosis only for individuals with cancer and age at censoring for unaffected mutation carriers.

**Table 2:** HLA class I and II statistical association in LSVH carrying the same *MLH1*:c.1528C > T PV. Alleles that showed statistical significance after gender adjustment (corrected *p*-value (q) < 0.05) are highlighted in gray. Abbreviations: HR: hazard ratio; CI: confidence interval.

Allele	Unadjusted HR (CI)	*p*-Value	q-Value	Adjusted HR (CI)	*p*-Value	q-Value
**HLA-A**						
A*01:01	1.31 (0.58–2.96)	0.515	0.890	1.78 (0.78–4.10)	0.173	0.384
A*02:01	1.76 (0.85–3.64)	0.130	0.495	1.40 (0.671–2.92)	0.371	0.571
A*02:05	1.19 (0.46–3.11)	0.717	0.934	0.96 (0.37–2.51)	0.932	0.932
A*11:01	5.08 (1.79–14.40)	0.002	0.043	4.14 (1.46–11.76)	0.008	0.062
A*23:01	1.46 (0.56–3.80)	0.441	0.838	1.84 (0.70–4.85)	0.218	0.436
A*24:02	2.21 (0.95–5.13)	0.066	0.414	2.58 (1.11–6.00)	0.028	0.113
A*26:01	2.23 (0.90–5.54)	0.084	0.414	3.32 (1.30–8.48)	0.012	0.062
A*29:01	1.21 (0.40–3.70)	0.738	0.934	1.84 (0.59–5.77)	0.296	0.537
A*29:02	1.27 (0.49–3.31)	0.627	0.934	1.23 (0.47–3.22)	0.667	0.852
A*30:01	0.83 (0.27–2.53)	0.737	0.934	0.81 (0.26–2.52)	0.722	0.852
A*30:02	1.59 (0.61–4.15)	0.341	0.721	1.13 (0.43–2.97)	0.808	0.898
A*30:04	1.01 (0.33–3.07)	0.992	0.992	1.11 (0.36–3.38)	0.861	0.906
A*32:01	0.60 (0.22–1.67)	0.327	0.721	0.44 (0.16–1.23)	0.117	0.334
A*33:03	2.44 (0.88–6.79)	0.087	0.414	3.84 (1.34–11.00)	0.012	0.062
A*34:02	1.13 (0.41–3.14)	0.816	0.943	1.70 (0.60–4.86)	0.322	0.537
A*43:01	1.52 (0.69–3.36)	0.298	0.721	2.04 (0.91–4.59)	0.083	0.275
A*68:01	0.92 (0.38–2.19)	0.843	0.943	0.79 (0.33–1.90)	0.602	0.852
A*68:02	0.98 (0.32–3.00)	0.978	0.992	1.22 (0.40–3.75)	0.724	0.852
HLA-A_Binned	1.56 (0.80–3.06)	0.193	0.610	1.67 (0.85–3.28)	0.134	0.336
**HLA-B**						
B*07:02	0.91 (0.39–2.12)	0.830	0.996	0.81 (0.37–1.88)	0.619	0.893
B*08:01	0.75 (0.30–1.90)	0.542	0.996	0.87 (0.34–2.22)	0.771	0.900
B*13:03	1.87 (0.62–5.62)	0.263	0.909	2.21 (0.73–6.66)	0.161	0.841
B*15:01	1.18 (0.43–3.21)	0.747	0.996	1.22 (0.45–3.31)	0.702	0.893
B*15:03	1.03 (0.47–2.27)	0.933	0.996	1.31 (0.59–2.94)	0.506	0.885
B*15:10	1.50 (0.64–3.47)	0.350	0.909	1.66 (0.71–3.88)	0.240	0.841
B*18:01	1.50 (0.68–3.28)	0.314	0.909	1.32 (0.60–2.91)	0.484	0.885
B*35:01	0.76 (0.30–1.93)	0.561	0.996	0.69 (0.27–1.75)	0.432	0.885
B*47:01	1.00 (0.37–2.73)	0.996	0.996	0.97 (0.36–2.64)	0.955	0.972
B*57:01	0.47 (0.16–1.39)	0.172	0.909	0.48 (0.16–1.44)	0.192	0.841
B*58:01	0.97 (0.38–2.48)	0.956	0.996	0.82 (0.32–2.10)	0.681	0.893
B*58:02	1.11 (0.52–2.37)	0.793	0.996	1.01 (0.47–2.17)	0.972	0.972
HLA-B_Binned	1.34 (0.78–2.31)	0.290	0.909	1.29 (0.75–2.23)	0.358	0.885
**HLA-C**						
C*02:10	0.87 (0.42–1.84)	0.725	0.725	0.82	0.596	0.596
C*04:01	1.28 (0.78–2.10)	0.335	0.600	1.40	0.189	0.474
C*05:01	1.76 (0.80–3.84)	0.158	0.600	1.43	0.378	0.478
C*07:01	1.15 (0.63–2.12)	0.650	0.725	1.41	0.284	0.474
C*07:02	1.52 (0.72–3.21)	0.276	0.600	1.35	0.430	0.478
C*07:04	1.63 (0.63–4.21)	0.316	0.600	1.74	0.253	0.474
C*16:01	1.29 (0.66–2.52)	0.457	0.600	1.35	0.386	0.478
C*17:01	1.39 (0.58–3.34)	0.467	0.600	1.79	0.203	0.474
HLA-C_Binned	1.55 (0.93–2.60)	0.096	0.600	1.57	0.085	0.426
**HLA-DRB1**						
DRB1*01:01	0.82 (0.25–2.70)	0.741	0.741	0.81 (0.24–2.66)	0.725	0.932
DRB1*03:01	1.32 (0.73–2.38)	0.354	0.659	1.30 (0.72–2.33)	0.389	0.612
DRB1*04:01	1.18 (0.65–2.14)	0.594	0.659	0.99 (0.54–1.81)	0.964	0.964
DRB1*10:01	3.16 (1.21–8.28)	0.019	0.107	3.03 (1.16–7.94)	0.024	0.089
DRB1*12:02	2.36 (1.14–4.91)	0.021	0.107	2.54 (1.22–5.29)	0.013	0.069
DRB1*13:01	1.27 (0.60–2.72)	0.532	0.659	1.12 (0.53–2.41)	0.762	0.932
DRB1*13:02	1.29 (0.53–3.14)	0.568	0.659	1.08 (0.44–2.63)	0.869	0.956
DRB1*15:01	1.76 (0.95–3.25)	0.071	0.237	1.76 (0.95–3.24)	0.072	0.158
DRB1*15:03	1.70 (0.90–3.22)	0.100	0.251	1.94 (1.02–3.69)	0.043	0.118
HLA-DRB1_Binned	1.26 (0.74–2.15)	0.399	0.659	1.35 (0.79–2.31)	0.273	0.501
**HLA-DRB3**						
DRB3*01:01	1.03 (0.58–1.84)	0.914	0.914	1.13 (0.63–2.03)	0.678	0.678
DRB3*02:02	0.81 (0.52–1.27)	0.369	0.531	0.79 (0.51–1.24)	0.310	0.516
DRB3*03:01	1.26 (0.73–2.18)	0.398	0.531	1.22 (0.71–2.09)	0.482	0.603
HLA-DRB3_Binned	2.11 (0.52–8.57)	0.299	0.531	2.70 (0.65–11.10)	0.170	0.424
**HLA-DRB4**						
DRB4*01:01	0.79 (0.52–1.20)	0.271	0.463	0.75 (0.49–1.14)	0.177	0.249
DRB4*01:03	0.85 (0.55–1.32)	0.463	0.463	0.77 (0.49–1.20)	0.249	0.249
HLA-DRB5						
DRB5*01:01	1.30 (0.78–2.17)	0.306	0.613	1.48 (0.88–2.48)	0.135	0.202
HLA-DRB5_Binned	0.92 (0.23–3.71)	0.905	0.905	1.02 (0.25–4.15)	0.973	0.973
**HLA-DQA1**						
DQA1*01:01	0.62 (0.34–1.15)	0.132	0.444	0.65 (0.35–1.19)	0.163	0.386
DQA1*01:03	0.71 (0.33–1.52)	0.374	0.534	0.64 (0.30–1.37)	0.247	0.386
DQA1*01:10	2.40 (0.73–7.89)	0.149	0.444	2.08 (0.63– 6.86)	0.229	0.386
DQA1*02:01	0.60 (0.36–0.99)	0.045	0.444	0.59 (0.36–0.97)	0.039	0.212
DQA1*03:01	0.80 (0.37–1.72)	0.571	0.635	0.65 (0.30–1.42)	0.281	0.386
DQA1*03:02	0.75 (0.43–1.29)	0.299	0.499	0.69 (0.40–1.20)	0.189	0.386
DQA1*04:01	0.84 (0.20–3.49)	0.814	0.814	0.92 (0.22–3.84)	0.913	0.913
DQA1*04:03	0.47 (0.11–1.95)	0.296	0.499	0.48 (0.12–2.01)	0.316	0.387
DQA1*05:01	0.71 (0.43–1.17)	0.178	0.444	0.73 (0.44–1.21)	0.220	0.386
DQA1*06:01	1.25 (0.61–2.59)	0.541	0.635	1.34 (0.65–2.77)	0.431	0.474
**HLA-DQB1**						
DQB1*02:01	0.74 (0.42–1.30)	0.298	0.537	0.74 (0.42–1.30)	0.291	0.485
DQB1*02:02	0.66 (0.40–1.09)	0.106	0.456	0.67 (0.40–1.10)	0.114	0.380
DQB1*03:01	0.97 (0.57–1.65)	0.902	0.902	0.95 (0.56–1.63)	0.858	0.858
DQB1*03:02	0.60 (0.33–1.07)	0.086	0.456	0.55 (0.30–0.99)	0.045	0.224
DQB1*04:02	0.75 (0.30–1.92)	0.553	0.647	0.69 (0.27–1.77)	0.445	0.635
DQB1*05:01	0.80 (0.42–1.54)	0.512	0.647	0.84 (0.44–1.62)	0.605	0.756
DQB1*06:03	0.77 (0.30–1.95)	0.575	0.647	0.82 (0.32–2.10)	0.680	0.756
DQB1*06:04	0.57 (0.20–1.61)	0.291	0.537	0.48 (0.17–1.36)	0.167	0.417
HLA-DQB1_Binned	0.61 (0.31–1.20)	0.152	0.456	0.65 (0.33–1.29)	0.219	0.438
**HLA-DPA1**						
DPA1*01:04	0.63 (0.25–1.56)	0.313	0.439	0.66 (0.27–1.65)	0.375	0.522
DPA1*02:01	0.76 (0.47–1.24)	0.277	0.439	0.77 (0.47–1.26)	0.304	0.522
DPA1*02:02	1.13 (0.76–1.68)	0.548	0.548	1.07 (0.72–1.60)	0.723	0.723
DPA1*02:07	0.54 (0.20–1.50)	0.238	0.439	0.65 (0.23–1.80)	0.404	0.522
DPA1*03:01	1.23 (0.69–2.17)	0.486	0.548	1.24 (0.70–2.20)	0.457	0.522
DPA1*04:01	1.84 (0.79–4.28)	0.156	0.439	2.26 (0.96–5.33)	0.062	0.247
HLA-DPA1_Binned	0.22 (0.03–1.58)	0.131	0.439	0.19 (0.03–1.39)	0.102	0.273
**HLA-DPB1**						
DPB1*02:01	1.14 (0.62–2.12)	0.672	0.928	1.35 (0.72–2.53)	0.36	0.844
DPB1*02:02	1.13 (0.50–2.54)	0.771	0.943	1.02 (0.45– 2.30)	0.96	0.986
DPB1*03:01	1.27 (0.66–2.44)	0.481	0.928	1.26 (0.65–2.43)	0.49	0.844
DPB1*04:01	0.89 (0.51–1.55)	0.675	0.928	0.88 (0.50–1.54)	0.66	0.877
DPB1*04:02	2.84 (1.18–6.80)	0.020	0.108	3.37 (1.39–8.16)	0.01	0.043
DPB1*05:01	1.64 (0.64–4.20)	0.302	0.928	1.57 (0.61–4.02)	0.35	0.844
DPB1*105:01	1.14 (0.70–1.86)	0.588	0.928	1.19 (0.73–1.94)	0.48	0.844
DPB1*13:01	0.94 (0.39–2.23)	0.886	0.975	0.93 (0.39–2.21)	0.86	0.986
DPB1*15:01	0.99 (0.39–2.54)	0.986	0.986	0.99 (0.39–2.54)	0.99	0.986
DPB1*558:01	0.57 (0.17–1.84)	0.346	0.928	0.72 (0.22–2.38)	0.59	0.877
HLA-DPB1_Binned	2.54 (1.33–4.82)	0.004	0.049	2.30 (1.21–4.38)	0.01	0.045

## Data Availability

The data analyzed during the current study are available from the corresponding author upon request.

## Supplementary Materials

The following supporting information can be downloaded at: https://www.mdpi.com/article/10.3390/jpm14060575/s1, Figure S1: A snapshot of a potential novel allele in the *HLA DRB3* locus.; Table S1: Frequencies of HLA class I and II alleles in heterozygous carriers of the *MLH1*:c1528C>T pathogenic variant.; Table S2: Allele frequencies of unique HLA alleles (alleles observed only once) in our study cohort (LSVH carrying the same *MLH1*:c.1528C>T PV) and other HLA-tested non-LSVH South African Populations.
