# Supplementary material for: Human Leukocyte Antigen-Allelic Variations May Influence the Age at Cancer Diagnosis in Lynch Syndrome
Source: J Pers Med. 2024 May 27;14(6):575. doi: 10.3390/jpm14060575 (PMC11204704; doi:10.3390/jpm14060575)
Supplement: Supplementary file 1 [file jpm-14-00575-s001.zip › Figure S1.pdf]

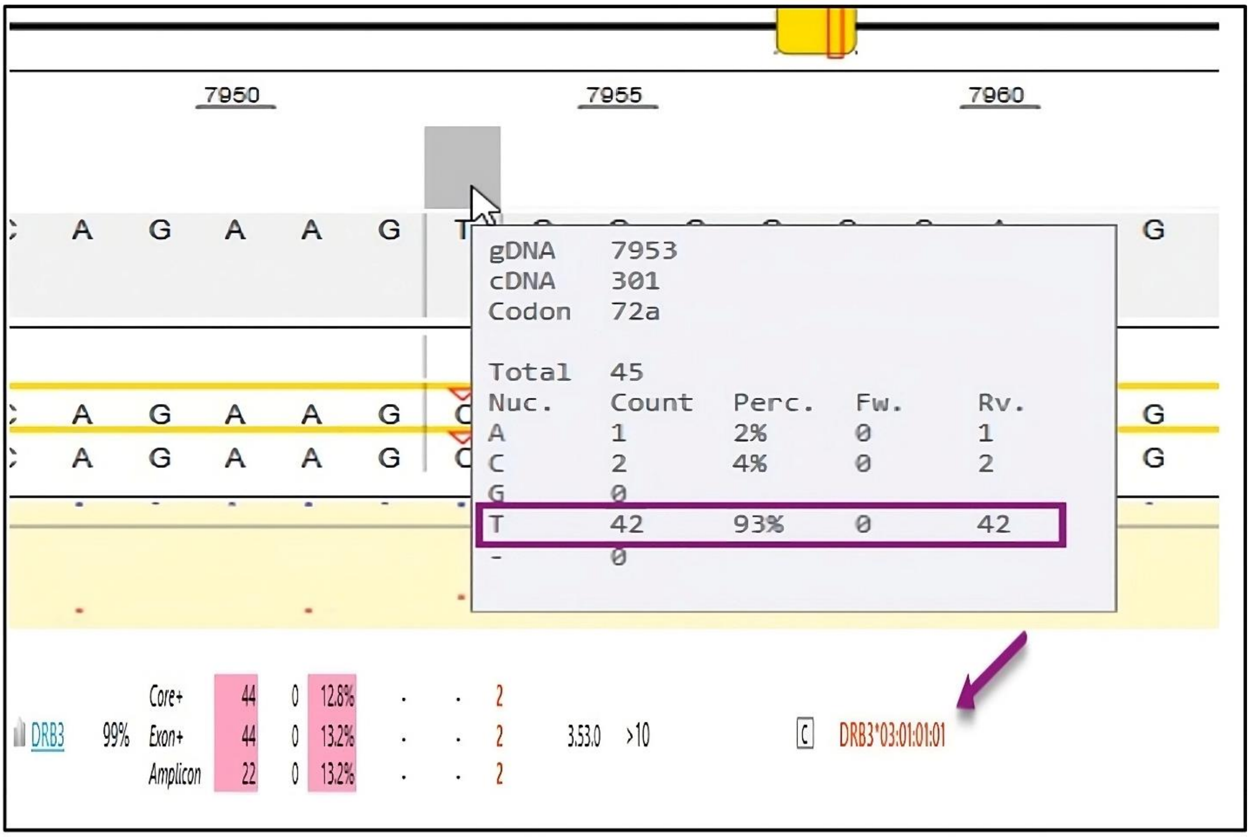

Supplementary Figure S1: A snapshot of a potential novel allele in the HLA DRB3 locus. A mismatch was identified in gDNA position 7953. Our sequencing results indicate a T in position 7953 while the closest allele *HLA-DRB3*03:01* has a C at this position. The T is present in 93% of the reads within the second exon of the HLA DRB3 locus, which will result in a change of the amino acid.
