# Supplementary material for: Human Leukocyte Antigen-Allelic Variations May Influence the Age at Cancer Diagnosis in Lynch Syndrome
Source: J Pers Med. 2024 May 27;14(6):575. doi: 10.3390/jpm14060575 (PMC11204704; doi:10.3390/jpm14060575)
Supplement: Supplementary file 1 [file jpm-14-00575-s001.zip › Supplementary Table S1.pdf]

Supplementary Table S1: Frequencies of HLA class I and II alleles in heterozygous carriers of the *MLH1*:c1528C>T pathogenic variant. Alleles with a count of less than 5 (rare alleles) are highlighted in blue. These rare alleles were grouped into a single category per locus for the downstream statistical analysis.

Locus	Allele	Counts	Frequencies	Percentage
		**HLA-A**		
A	A*01:01	13	0.071	7.1
A	A*01:23	1	0.005	0.5
A	A*02:01	15	0.082	8.2
A	A*02:02	3	0.016	1.6
A	A*02:05	7	0.038	3.8
A	A*02:11	1	0.005	0.5
A	A*02:24	1	0.005	0.5
A	A*03:01	18	0.099	9.9
A	A*11:01	5	0.027	2.7
A	A*23:01	7	0.038	3.8
A	A*24:02	9	0.049	4.9
A	A*24:07	4	0.022	2.2
A	A*26:01	5	0.027	2.7
A	A*29:01	7	0.038	3.8
A	A*29:02	7	0.038	3.8
A	A*30:01	5	0.027	2.7
A	A*30:02	6	0.033	3.3
A	A*30:04	5	0.027	2.7
A	A*31:06	2	0.011	1.1
A	A*32:01	10	0.055	5.5
A	A*33:01	1	0.005	0.5
A	A*33:03	6	0.033	3.3
A	A*34:01	3	0.016	1.6
A	A*34:02	5	0.027	2.7
A	A*36:01	1	0.005	0.5
A	A*43:01	12	0.066	6.6
A	A*66:01	2	0.011	1.1
A	A*68:01	10	0.055	5.5
A	A*68:02	6	0.033	3.3
A	A*68:27	4	0.022	2.2
A	A*74:01	1	0.005	0.5
		**HLA-B**		
B	B*07:02	11	0.060	6.0
B	B*07:05	3	0.016	1.6
B	B*08:01	9	0.049	4.9
B	B*13:01	1	0.005	0.5
B	B*13:02	4	0.022	2.2
B	B*13:03	4	0.022	2.2
B	B*14:01	1	0.005	0.5
B	B*14:03	1	0.005	0.5
B	B*15:01	5	0.027	2.7
B	B*15:02	2	0.011	1.1
B	B*15:03	11	0.060	6.0
B	B*15:10	8	0.044	4.4
B	B*15:13	2	0.011	1.1
B	B*15:17	2	0.011	1.1
B	B*18:01	10	0.055	5.5

B	B*27:05	3	0.016	1.6
B	B*35:01	7	0.038	3.8
B	B*35:02	1	0.005	0.5
B	B*35:05	4	0.022	2.2
B	B*39:06	1	0.005	0.5
B	B*39:10	2	0.011	1.1
B	B*40:01	2	0.011	1.1
B	B*41:01	4	0.022	2.2
B	B*42:01	1	0.005	0.5
B	B*44:02	7	0.038	3.8
B	B*44:03	21	0.115	11.5
B	B*44:04	2	0.011	1.1
B	B*45:01	4	0.022	2.2
B	B*47:01	6	0.033	3.3
B	B*51:01	4	0.022	2.2
B	B*51:02	3	0.016	1.6
B	B*53:01	1	0.005	0.5
B	B*56:01	3	0.016	1.6
B	B*57:01	7	0.038	3.8
B	B*57:03	3	0.016	1.6
B	B*58:01	7	0.038	3.8
B	B*58:02	13	0.071	7.1
B	B*81:01	1	0.005	0.5
**HLA-C**				
C	C*01:02	3	0.016	1.6
C	C*02:02	1	0.005	0.5
C	C*02:10	12	0.066	6.6
C	C*03:02	2	0.011	1.1
C	C*03:03	3	0.016	1.6
C	C*03:04	3	0.016	1.6
C	C*04:01	36	0.198	19.8
C	C*05:01	8	0.044	4.4
C	C*06:02	37	0.203	20.3
C	C*07:01	22	0.121	12.1
C	C*07:02	11	0.060	6.0
C	C*07:04	5	0.027	2.7
C	C*08:01	4	0.022	2.2
C	C*08:04	2	0.011	1.1
C	C*12:02	2	0.011	1.1
C	C*14:02	3	0.016	1.6
C	C*15:02	2	0.011	1.1
C	C*15:05	3	0.016	1.6
C	C*16:01	13	0.071	7.1
C	C*17:01	7	0.038	3.8
C	C*18:01	2	0.011	1.1
**HLA-DRB1**				
DRB1	DRB1*01:01	5	0.027	2.7
DRB1	DRB1*01:02	3	0.016	1.6
DRB1	DRB1*03:01	21	0.115	11.5
DRB1	DRB1*03:02	1	0.005	0.5
DRB1	DRB1*04:01	21	0.115	11.5
DRB1	DRB1*04:03	1	0.005	0.5
DRB1	DRB1*04:04	4	0.022	2.2
DRB1	DRB1*04:129	1	0.005	0.5
DRB1	DRB1*07:01	34	0.187	18.7

DRB1	DRB1*08:03	1	0.005	0.5
DRB1	DRB1*08:04	4	0.022	2.2
DRB1	DRB1*08:12	1	0.005	0.5
DRB1	DRB1*09:01	2	0.011	1.1
DRB1	DRB1*10:01	5	0.027	2.7
DRB1	DRB1*11:01	3	0.016	1.6
DRB1	DRB1*11:02	1	0.005	0.5
DRB1	DRB1*11:03	1	0.005	0.5
DRB1	DRB1*11:04	1	0.005	0.5
DRB1	DRB1*12:01	1	0.005	0.5
DRB1	DRB1*12:02	10	0.055	5.5
DRB1	DRB1*13:01	11	0.060	6.0
DRB1	DRB1*13:02	8	0.044	4.4
DRB1	DRB1*13:03	2	0.011	1.1
DRB1	DRB1*14:04	1	0.005	0.5
DRB1	DRB1*14:54	3	0.016	1.6
DRB1	DRB1*15:01	18	0.099	9.9
DRB1	DRB1*15:02	2	0.011	1.1
DRB1	DRB1*15:03	16	0.088	8.8
**HLA-DRB3**				
DRB3	DRB3*01:01	17	0.093	9.3
DRB3	DRB3*02	1	0.005	0.5
DRB3	DRB3*02:02	28	0.154	15.4
DRB3	DRB3*03:01	17	0.093	9.3
DRB3	DRB3*03:Novel	1	0.005	0.5
DRB3	DRB3*NNNN	118	0.648	64.8
**HLA-DRB4**				
DRB4	DRB4*01:01	34	0.187	18.7
DRB4	DRB4*01:03	29	0.159	15.9
DRB4	DRB4*NNNN	119	0.654	65.4
**HLA-DRB5**				
DRB5	DRB5*01:01	18	0.099	9.9
DRB5	DRB5*01:08	2	0.011	1.1
DRB5	DRB5*NNNN	162	0.890	89.0
**HLA-DQA1**				
DQA1	DQA1*01:01	19	0.104	10.4
DQA1	DQA1*01:02	44	0.242	24.2
DQA1	DQA1*01:03	10	0.055	5.5
DQA1	DQA1*01:10	3	0.016	1.6
DQA1	DQA1*02:01	32	0.176	17.6
DQA1	DQA1*03:01	9	0.049	4.9
DQA1	DQA1*03:02	22	0.242	24.2
DQA1	DQA1*04:03	3	0.033	3.3
DQA1	DQA1*04:01	2	0.022	2.2
DQA1	DQA1*05:01	29	0.319	31.9
DQA1	DQA1*06:01	9	0.099	9.9
**HLA- DQB1**				
DQB1	DQB1*02:01	20	0.110	11.0
DQB1	DQB1*02:02	31	0.170	17.0
DQB1	DQB1*03:01	23	0.126	12.6
DQB1	DQB1*03:02	20	0.110	11.0
DQB1	DQB1*03:03	3	0.016	1.6
DQB1	DQB1*03:19	3	0.016	1.6
DQB1	DQB1*04:02	6	0.066	6.6
DQB1	DQB1*05:01	15	0.165	16.5

DQB1	DQB1*05:03	4	0.044	4.4
DQB1	DQB1*06:01	2	0.022	2.2
DQB1	DQB1*06:02	40	0.440	44.0
DQB1	DQB1*06:03	7	0.077	7.7
DQB1	DQB1*06:04	6	0.066	6.6
DQB1	DQB1*06:09	2	0.022	2.2
**HLA-DPA1**				
DPA1	DPA1*01:03	70	0.385	38.5
DPA1	DPA1*01:04	7	0.038	3.8
DPA1	DPA1*01:05	1	0.005	0.5
DPA1	DPA1*02:01	29	0.159	15.9
DPA1	DPA1*02:02	45	0.247	24.7
DPA1	DPA1*02:07	6	0.033	3.3
DPA1	DPA1*02:09	2	0.022	2.2
DPA1	DPA1*03:01	16	0.176	17.6
DPA1	DPA1*04:01	6	0.066	6.6
**HLA- DPB1**				
DPB1	DPB1*01:01	43	0.236	23.6
DPB1	DPB1*02:01	15	0.082	8.2
DPB1	DPB1*02:02	8	0.044	4.4
DPB1	DPB1*03:01	15	0.082	8.2
DPB1	DPB1*04:01	24	0.132	13.2
DPB1	DPB1*04:02	6	0.033	3.3
DPB1	DPB1*05:01	5	0.027	2.7
DPB1	DPB1*09:01	3	0.016	1.6
DPB1	DPB1*10:01	1	0.005	0.5
DPB1	DPB1*105:01	34	0.187	18.7
DPB1	DPB1*106:01	1	0.005	0.5
DPB1	DPB1*13:01	8	0.044	4.4
DPB1	DPB1*15:01	6	0.033	3.3
DPB1	DPB1*18:01	1	0.005	0.5
DPB1	DPB1*20:01	1	0.005	0.5
DPB1	DPB1*26:01	2	0.011	1.1
DPB1	DPB1*28:01	1	0.005	0.5
DPB1	DPB1*296:01	1	0.005	0.5
DPB1	DPB1*55:01	2	0.011	1.1
DPB1	DPB1*558:01	5	0.027	2.7
