# Supplementary material for: Human Leukocyte Antigen-Allelic Variations May Influence the Age at Cancer Diagnosis in Lynch Syndrome
Source: J Pers Med. 2024 May 27;14(6):575. doi: 10.3390/jpm14060575 (PMC11204704; doi:10.3390/jpm14060575)
Supplement: Supplementary file 1 [file jpm-14-00575-s001.zip › Supplementary Table S2.pdf]

Supplementary Table S2: Allele frequencies of unique HLA alleles (alleles observed only once) in our study cohort (LSVH carrying the same *MLH1*:c.1528C>T PV) and other HLA-tested non-LSVH South African Populations. Note: Crosses indicate that the allele was not typed in the specific population.

**Allele**	**SA LSVH**	**SA Worcester**	**SA Black**	**SA Caucasians**	**SA Natal Zulu**	**SA Natal Tamil**	**SA Indian population**	**SA Mixed ancestry**	**SA Limpopo Venda**
A*01:23	0.005	0.016	X	X	X	X	X	X	X
A*02:11	0.005	0.006	X	X	0.000	0.130	0.040	0.020	X
A*02:24	0.005	0.000	X	X	X	X	X	X	X
A*33:01	0.005	0.006	X	0.010	0.000	X	X	X	X
A*36:01	0.005	0.006	0.007	0.000	0.000	X	X	X	X
A*74:01	0.005	0.031	X	0.000	X	X	X	X	X
B*13:01	0.005	X	X	X	0.000	0.061	X	X	X
B*14:01	0.005	0.009	X	0.005	0.04	X	X	X	X
B*14:03	0.005	X	X	X	X	X	X	X	X
B*35:02	0.005	0.006	X	X	0.000	X	X	X	X
B*39:06	0.005	X	X	X	0.000	X	X	0.010	X
B*42:01	0.005	0.022	X	0.000	0.12	X	X	X	X
B*53:01	0.005	0.025	X	0.000	0.015	X	X	X	X
B*81:01	0.005	0.016	0.039	X	0.035	X	X	0.010	X
C*02:02	0.005	0.028	0.025	0.069	X	0.010	X	0.040	X
DPA1*01:05	0.005	X	X	X	X	X	X	X	X
DPB1*10:01	0.005	X	X	X	X	X	X	X	X
DPB1*106:01	0.005	0.006	0.007	X	X	X	X	X	X
DPB1*18:01	0.005	0.041	0.056	X	X	X	X	X	X
DPB1*20:01	0.005	X	X	X	X	X	X	X	X
DPB1*28:01	0.005	0.006	X	X	X	X	X	X	X
DPB1*296:01	0.005	X	X	X	X	X	X	X	X
DRB1*03:02	0.005	0.041	0.092	0.005	X	X	X	X	0.090
DRB1*04:03	0.005	0.006	X	0.016	X	X	X	X	X
DRB1*04:129	0.005	X	X	X	X	X	X	X	X
DRB1*08:03	0.005	0.003	X	X	X	X	X	X	X
DRB1*08:12	0.005	X	X	X	X	X	X	X	X
DRB1*11:02	0.005	0.025	0.070	0.000	X	X	X	X	X
DRB1*11:03	0.005	X	X	X	X	X	X	X	X
DRB1*11:04	0.005	0.006	X	0.037	X	X	X	X	X
DRB1*12:01	0.005	0.025	0.028	0.011	X	X	X	X	X
DRB1*14:04	0.005	0.016	X	0.005	X	X	X	X	X
DRB3*02	0.005	X	X	X	X	X	X	X	X
DRB3*03:Novel	0.005	X	X	X	X	X	X	X	X
